# Identification of broken conductor faults in interconnected transmission systems based on discrete wavelet transform

**DOI:** 10.1371/journal.pone.0296773

**Published:** 2024-01-12

**Authors:** Basem Abd-Elhamed Rashad, Doaa K. Ibrahim, Mahmoud I. Gilany, Ahmed Sayed Abdelhamid, Wael Abdelfattah

**Affiliations:** 1 Department of Electrical Power and Machines Engineering, The Higher Institute of Engineering at El-Shorouk City, El-Shorouk Academy, Cairo, Egypt; 2 Department of Electrical Power Engineering, Faculty of Engineering, Cairo University, Giza, Egypt; Vardhaman College of Engineering, INDIA

## Abstract

Interconnected transmission systems are increasingly spreading out in HV networks to enhance system efficiency, decrease reserve capacity, and improve service reliability. However, the protection of multi-terminal lines against Broken Conductor Fault (BCF) imposes significant difficulties in such networks as the conventional distance relays cannot detect BCF, as the BCF is not associated with a significant increase in current or reduction in voltage Traditionally, the earth fault relays in transmission lines may detect such fault; Nonetheless, it suffers from a long delay time. Moreover, many of the nearby earth fault relays detect the BCF causing unnecessary trips and badly affecting the system stability. In this article, a novel single-end scheme based on extracting transient features from current signals by discrete wavelet transform (DWT) is proposed for detecting BCFs in interconnected HV transmission systems. The suggested scheme unit (SSU) is capable of accurately detecting all types of BCFs and shunt high impedance faults (SHIFs). It also adaptively calculates the applied threshold values. The accurate selectivity in multi-terminal lines is achieved based on a fault directional element by analyzing transient power polarity. The SSU discriminates between internal/external faults effectively utilizing the time difference observed between the first spikes of aerial and ground modes in the current signals. Different fault scenarios have been simulated on the IEEE 9-Bus, 230 kV interconnected system. The achieved results confirm the effectiveness, robustness, and reliability of SSU in detecting correctly BCFs as well as the SHIFs within only 24.5 ms. The SSU has confirmed its capability to be implemented in interconnected systems without any requirement for communication or synchronization between the SSU installed in multi-terminal lines.

## Introduction

The increasing focus on interconnected transmission systems reflects the ongoing advancements in global economic and social issues. Considering the investment savings and technical benefits over double-ended systems, multi-terminal transmission systems such as interconnected systems and teed connection transmission lines are increasingly performing in high voltage (HV) and ultra-high voltage power networks [1, 2]. However, the protection of such multi-terminal transmission systems is more complex compared to double-ended systems [3]. The problem is that many subsystems and fault locations are relatively dispersed. Furthermore, the consequences of malfunctions arising within individual local subsystems can spread via physical connections to neighboring subsystems, resulting in a decline in overall system efficiency or potentially causing false tripping. Protecting multi-terminal lines imposes serious challenges with fault detection and isolation [4]. Several conventional digital techniques, such as differential relaying schemes, directional comparison techniques, and distance protection, can potentially be applied in multi-terminal transmission systems protection. However, the main drawback of these schemes is that they require communication channels to transmit measurement data from terminals to either the main processing unit or to other terminals. In fact, a communication link is established for each phase which is very expensive [5–7]. Thus, there is a need for effective local solutions with reduced costs as they do not rely on communication systems.

Detecting and isolating faults reliably is the main target for any transmission line protection. Such faults are typically classified into series and shunt faults. Undesirable contact between power conductors and non-conductive surfaces like tree branches is the main cause for shunt high impedance faults (SHIFs) [8–10]. The condition mentioned above poses significant challenges for conventional protection relays. The series HIFs include broken conductor faults (BCFs), and their causes can be classified as described in Ref. [11]:

- Unintentional tripping of breaker operation,
- Broken (open) Bridle case and,
- Direct breaking of the line conductor.

Detection of BCFs is considered a real challenge as BCFs do not cause a significant change in current or voltage due to their minimal impact on current or voltage levels, which makes the distance relay, which are the primary protection of transmission lines (TLs) unable to detect such faults. Traditionally, a BCF in TL will continue until the earth fault relay, as a backup protection scheme, detects it after a long delay time [12]. It is worth mentioning that many of the nearby earth fault relays will detect BCF as the unbalance condition is widely spread. Unnecessary trips are experienced during the clearance of such faults. Consequently, opening a conductor in TL will adversely affect the system’s stability. The operation of the system in only two phases results in a decrease in the power transmitted through the line, causing overload in the healthy phases, and affecting their insulators. Besides, serious concerns regarding public safety, property damage, electric shocks, and fire hazards associated with BCFs make it crucial for utilities and society to identify such faults promptly [13].

Several fault detection methodologies have been reported in the literature to protect HV networks. Only a limited number of them have been implemented for the protection of BCFs, including the techniques involving negative sequence components and negative to positive current sequence techniques, as outlined in Refs. [14, 15]. These techniques have some limitations for practical applications, such as using several threshold values, where estimating their proper values for practical systems is a real challenge. Artificial intelligence-based protection schemes are also suggested in several studies, as proposed in Refs. [16–19]. In Ref. [17], a protection scheme is proposed based on an artificial neural network (ANN) to protect the HV transmission lines against Open Conductor Faults (OCFs), while in Ref. [18], a K-nearest neighbor-based protection scheme has been introduced for the detection and classification of all open conductor faults utilizing the fundamental component of current signals. It has also been developed in Ref. [19] to employ the fuzzy inference approach to implement unearthed open conductor fault detection in a double-circuit transmission line. The effectiveness of artificial intelligence-based protection schemes is severely reliant on their architecture and training, both of which may increase to their sophistication and cost. In contrast, academic literature has extensively documented various algorithms over the past two decades, as mentioned in Refs. [20, 21], that offer precise techniques for identifying BCFs in distribution networks. For example, in Ref. [20], voltage unbalance is utilized as an indicator of performance to identify OCFs. This requires the installation of sensors along the feeder and using advanced communication links between the sensors. Meanwhile, Ref. [21], presents a current detection method to identify open phases dependent on the current imbalance. However, this method is only appropriate for distribution networks that are grounded. It is worth mentioning that there is still a gap in the detection and classification of BCFs in HV transmission lines, most of these methods that have been introduced in the literature do not offer comprehensive coverage of the BCFs problem. Thus, a necessity for **novel** and **effective** schemes is **vital**.

**Fig 1:**
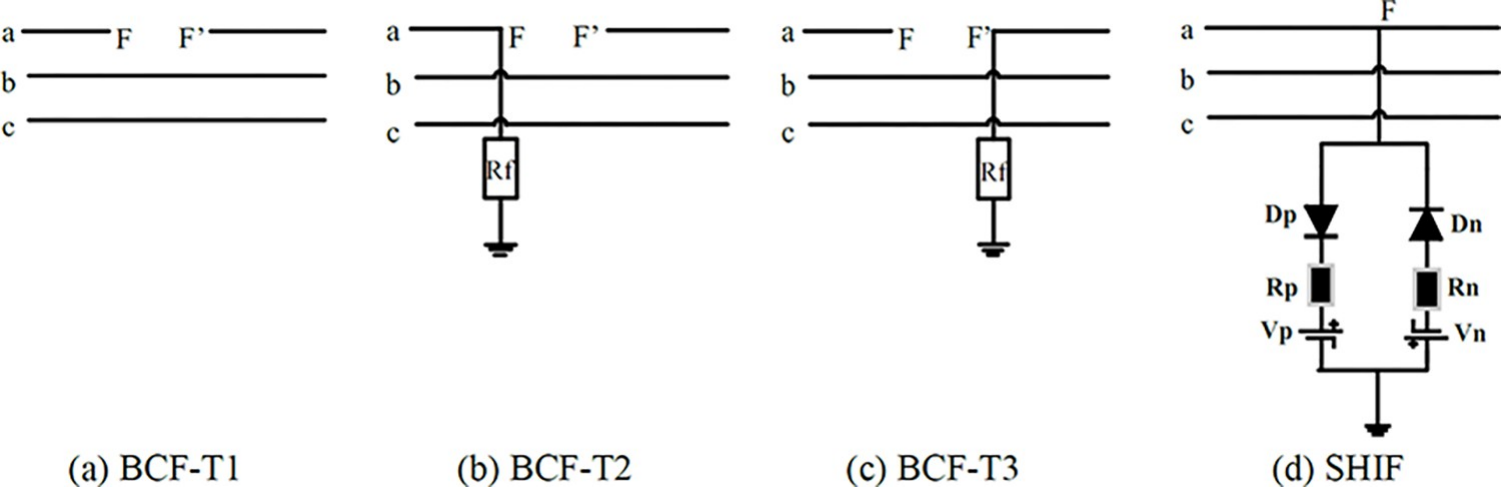
Presentation for different studied faults.

As described before, the most challenging problem related to **BCFs** is that the traditional distance relays do not detect them. Besides, they may be detected by earth relays in the interconnected network after an extended delay. Consequently, it will be cleared with multiple earth fault relays in the network, which badly affects system stability and performance. In this paper, a novel single-end measurement scheme is proposed to detect and isolate different types of BCFs and correctly discriminate the faulty line in interconnected HV networks. The main objective of the proposed scheme is to quickly decide the faulty line, whatever the fault type (BCF or SHIF). Fig 1 summarizes the studied fault types in this paper, where phase A is the faulty phase in such presented faults. In the broken conductor fault, BCF-T1, the protected TL has a broken conductor from both ends, as illustrated in Fig 1(A). The following two types (BCF-T2 and BCF-T3, presented in Fig 1(B) and 1(C) denote the representation of a BCF from one end-side in conjunction with an earth fault from the other end-side, where the earth fault in BCF-T3 is located on the relay end-side while it is on the opposite end-side in BCF-T2. The shunt HIF (without opening the circuit) is illustrated in Fig 1(D), where the shunt HIF model is constructed by assuming the arcing in sandy soil, as discussed in Ref. [22].

The discrete wavelet transform (DWT) is employed in the proposed single-end measurement scheme, which provides the perfect time-frequency localization ability to extract the fault transient characteristics to correctly detect different BCFs in HV interconnected transmission systems and discriminate BCFs from shunt HIFs. The suggested scheme is designed to be employed in an interconnected IEEE-9 bus system configuration without communicating or synchronizing between the SSUs at the two ends of each protected line. Each SSU can locally check whether the detected fault is internal/external to ensure proper selectivity. The simulation results demonstrate that the suggested scheme can adequately detect and isolate all BCFs and SHIFs in interconnected HV networks.

**The main contributions of the paper are listed as follows**:

■ To investigate the protection of interconnected transmission networks against Broken Conductor Fault (**BCF**).
■ To introduce the novel single-end scheme for accurately identifying all types of **BCFs** and shunt high impedance faults (**SHIFs**) in HV multi-terminal lines.
■ Accurate selectivity of the faulty line in multi-terminal lines is achieved using transient power polarity and the time difference observed between the initial spikes of aerial/ground modes of current signals.
■ To accurately detect **BCFs**, the suggested scheme unit (**SSU**) uses single-level DWT on local current signals and adaptively calculates threshold values.
■ The achieved results confirm the effectiveness, robustness, and reliability of SSU in detecting **BCFs** and discriminate/identify correctly between **BCFs** and **SHIFs**.

The structure of the rest of the paper is as follows. **Section “Background for applying DWT”,** provides a comprehensive overview of the background and rationale behind the application of the DWT. **Section “Description of the suggested scheme unit (SSU)”,** investigates the description of the Suggested Scheme Unit (**SSU**) as well as its proposed fault identification scheme and faulty line discrimination algorithm are fulfilled. **Section “Testing the performance of the suggested scheme unit (SSU)”,** evaluates the SSU’s performance in identifying faults and discriminating faulty lines using ATP/EMTP. Finally, **Section “General features of the suggested scheme”,** investigates the benefits of the suggested scheme, and **Section “Conclusions”,** introduces the paper’s conclusion.

## Background for applying DWT

When analyzing non-stationary signals, employing mathematical transforms such as Fourier Transform (FT) or Short-Time Fourier Transform (STFT) for time-frequency analysis may yield less satisfactory results in comparison to the capabilities offered by wavelet transform (WT). This is attributed to the WT’s capacity to conduct multi-resolution analysis, providing superior outcomes. Several WT applications have been employed in research studies for TLs fault detection, such as singular wavelet entropy, DWT, and wavelet packet transform [23–25]. The algorithm introduced in Ref. [26] depends only on the high-frequency components, which leads to misclassification between low impedance faults (LIFs) and SHIFs. In contrast, the proposed one in Ref. [27] depends only on low-frequency components, so it failed to detect SHIFs accurately. The combination of low and high-frequency components is proposed in Ref. [28] with fixed threshold values. Most of the studies based on WT were interested only in detecting shunt HIFs, **not BCFs**. So far, little attention has been paid to detecting BCFs cases precisely and accurately, the broken conductor from the two sides (BCF-T1) in the HV interconnected transmission systems area.

Generally, DWT can be applied to analyze a signal by recursively filtering it with high-pass and low-pass filter pairs of equal bandwidths. To achieve an additional level of decomposition, the cascaded procedure is implemented, whereby the output signal from the low-frequency band serves as the input for the subsequent decomposition level. In this process, the details of the signal are represented by the low-scale, high-frequency components that have been filtered through the high-pass filters. For analyzing the signal *x*[*n*] at the first level, wavelet coefficients can be decided mathematically as follows, where *A*1_*low*_[*k*] and *D*1_*high*_[*k*] is the approximation and detailed wavelet components, respectively:

$${A1}_{low}[k]=\sum_{n=-\infty }^{n=\infty }x[n]\times L[2k-n]$$ (1)

$${D1}_{high}[k]=\sum_{n=-\infty }^{n=\infty }x[n]\times h[2k-n]$$ (2)

As will be described later, DWT is applied in this paper to decompose the current signals at the first level into approximation and detailed wavelet components to enhance the detection/classification of different types of BCFs using indices estimated based on the maximum absolute of approximation (*M*−*Index*) coefficients and the difference of the detail coefficients (*H*−*Index*) of local current signals. 200 kHz sampling rate at 50 Hz (4000 samples/cycle) is employed for this single-level DWT decomposition. Consequently, in the frequency domain, the range of the 1^st^ detail coefficient (D1) is from 50 kHz to 100 kHz, while the range of the 1^st^ approximation coefficient (A1) is from 0 kHz to 50 kHz.

### Selecting the mother wavelet

The selection of the appropriate mother wavelet for implementation is contingent upon the nature of the application requirements for fault detection and classification. This ensures that the chosen mother wavelet (optimal choice) aligns with the nature requirements and characteristics of the particular application at hand, as outlined in refs. [29–31]. For the effective detection/classification of BCFs in the SSU, a suitable choice of mother wavelet is crucial. The selected mother wavelet should possess certain attributes that indicate a good correlation with the fault signal, characterized by the following attributes:

■ The highest magnitude of the first level of the detailed coefficients. The absolute sum (*S*_*abs*_(*k*)) for the current detail coefficient (*D*1) of a discrete-time signal is represented by Eq (3), where *n* represents the order of a sample within a sliding window, and *N* denotes the overall number of samples within a cycle, covering a power cycle of 20 **ms**.

$$S_{abs}(k)=\sum_{n=k-N+1}^{k}\left|D1(n)\right|$$ (3)

■ The smallest error (*ε*_*err*_) between the original signal (*x*) and the reconstructed signal (*x*′) to ensure perfect reconstruction. The error (*ε*_*err*_) is expressed by Eq (4), where the Euclidean length of the error vector (*ε*_*err*_) is calculated based on the norm function.

$$\varepsilon_{err}=||x-x^{\prime }||=\sqrt{\sum |x(n)-x^{\prime }(n){|}^{2}}$$ (4)

**Fig 2:**
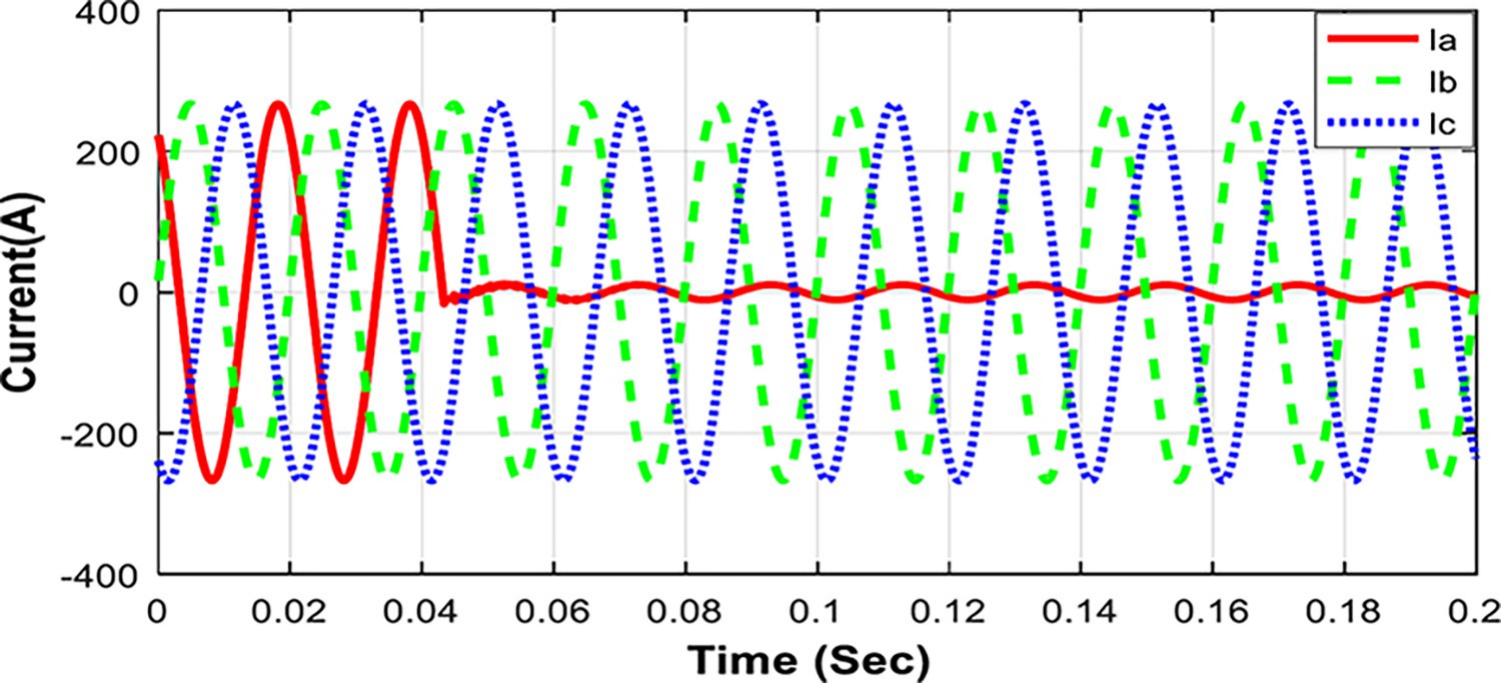
Current waveforms (*Ia*, *Ib*, *Ic*) of BCF fault (Type-1) at 30 km from SSU location.

Three wavelet families: Coiflets, Daubechies, and Symlets, are examined here to choose the most suitable one. Type-1 of a BCF on phase-A is simulated to occur 30 km from the SSU location. The measured current signals are shown in Fig 2. An extensive comparison is carried out among different orders (from 1 to 5) of these mother wavelet functions in terms of the 1^st^ level current detail coefficients as the sum of the absolute values (*S*_*abs*_(*k*)) and the smallest error (*ε*_*err*_) for the current phase A, as described in Table 1. The results emphasize the suitability of the Daubechies family as their related transforms are accurate, fast, and stable. As illustrated in Table 1, The selected mother wavelet (db1) indicates a reputable correlation with the faulted signal and ensures the best performance according to the two general conditions mentioned above: the maximum absolute sum (*S*_*abs*_(*k*)) of the 1^st^ level for current detail coefficients and, simultaneously, the smallest error between the reconstructed and the original signals. Thus, db1 is selected here to carry out the proposed detection procedure. Moreover, a single level of decomposition will be used only for the detection technique, which indicates that SSU will have less computing requirements compared to the multilevel decomposition approach utilized in numerous published research studies, such as Ref. [32].

**Table 1:** Comparative performance of different mother wavelets for detecting different BCFs.

Mother Wavelet	*S*_*abs*_(*k*)×103					*ε*_*err*_×10−12				
Mother Order	1	2	3	4	5	1	2	3	4	5
Daubechies (Db)	1.387	0.745	0.592	0.575	0.608	2.9011	3.3306	23.231	9.1675	16.5056
Coiflets (Coif)	0.925	0.632	0.577	0.558	0.550	3.9589	23.574	8.5249	33.618	51.3751
Symlets (Sym)	1.053	0.722	0.582	0.608	0.477	2.9822	3.3309	20.331	7.1614	8.11623

## Description of the suggested scheme unit (SSU)

**Fig 3:**
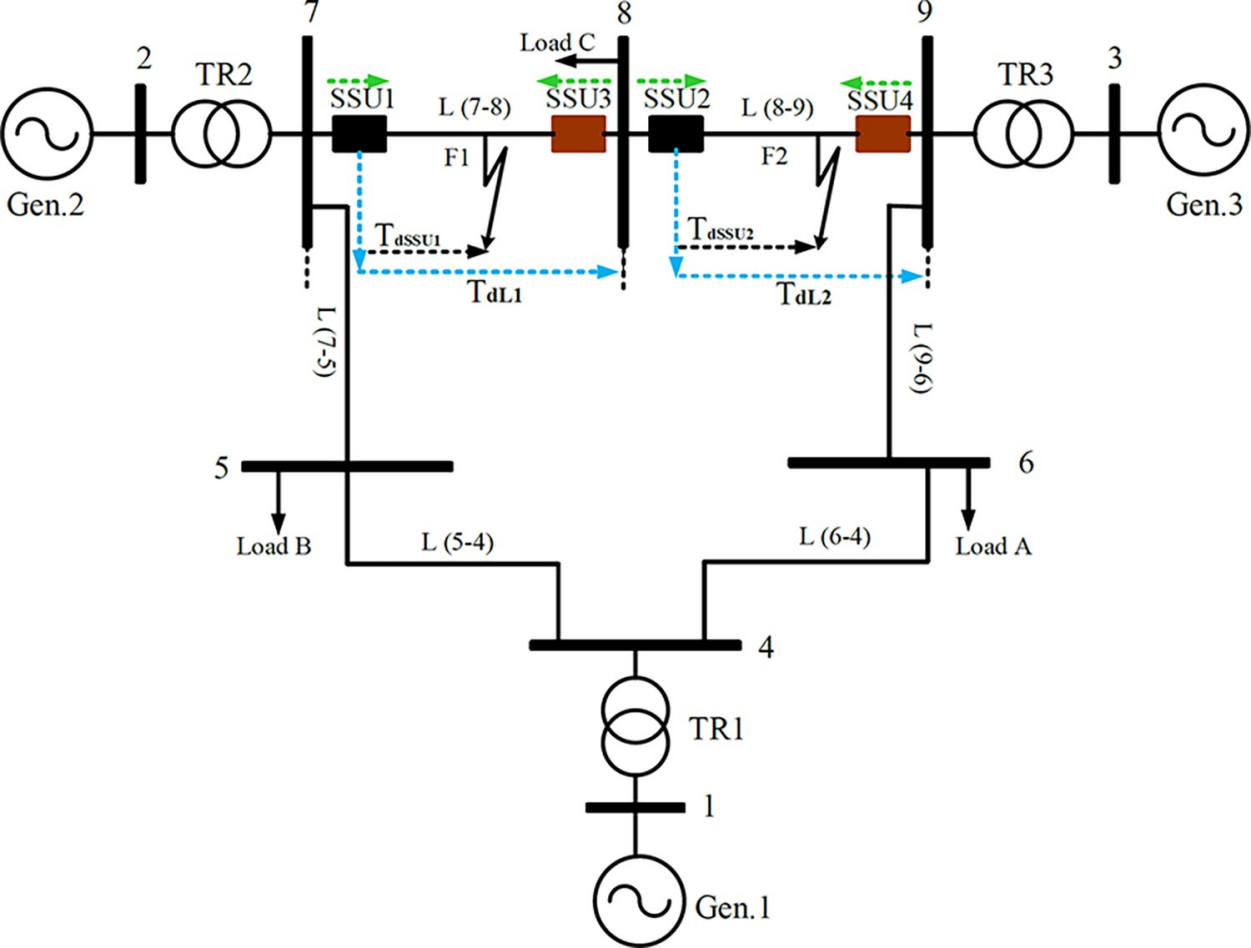
The interconnected transmission IEEE 9-bus system single line diagram.

**Fig 4:**
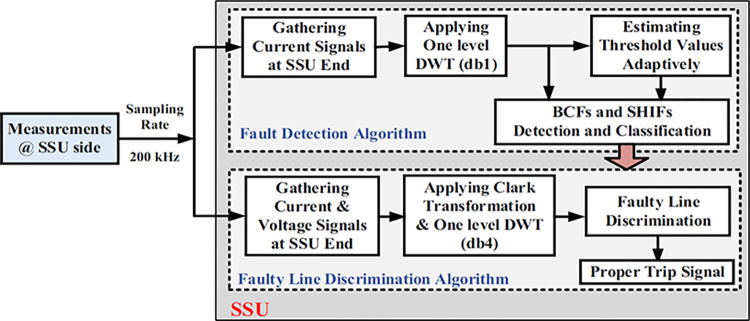
Overall structure of the suggested scheme unit (SSU).

The suggested scheme unit (SSU) is designed to identify and isolate BCFs in HV interconnected transmission systems such as the 230 kV, IEEE 9-bus system shown in Fig 3. To describe the SSU, consider the line L (7–8) as an example, where SSU1 & SSU3 are installed at the line terminals without any communication or synchronization between them. Upon capturing the voltage and current signals, each SSU will execute two algorithms (refer to Fig 4):

■ The first one is the fault detection algorithm. It is designed not only to detect but also to discriminate between different BCFs and shunt HIFs. It is based on DWT decomposition for current signals by applying db1. It estimates adaptively the updated threshold values to avoid any impact of changing system parameters. The second algorithm will only be activated upon detecting any BCFs or SHIFs.
■ The second algorithm is the faulty line discrimination algorithm. As stated, BCFs may be detected by multiple earth fault relays in the interconnected network after a long delay. This second algorithm aims to solve this problem and ensure good selectivity by quickly determining the faulty line. The algorithm is based on applying Clark transformation and getting the first level DWT decomposition for aerial modes of voltage and current signals using db4. The fault direction is decided by examining the polarity of the transient power. db4 mother wavelet is chosen in this case to meet the demands of rapid identification of transient signals and to yield satisfactory outcomes in capturing the arrival time of incident traveling waves, in contrast to db1 mother wavelet. The db4 mother wavelet possesses more desirable properties, such as multiple vanishing moments and improved frequency resolution, as opposed to db1 mother wavelet, which only has a single vanishing moment and exhibits poorer frequency resolution.

### Fault detection algorithm

This suggested fault detection algorithm depends on the decomposed current approximation coefficients of low-frequency components (1^st^ A1 ranges from 0 kHz to 50 kHz) and detail coefficients of high-frequency component (1^st^ D1 ranges from 50 kHz to 100 kHz) for the three-phase current signals (*ia*, *ib*, and *ic*), and ground current signal (*ig*), where *ig* = (*ia*+*ib*+*ic*) based on DWT, which is applied in the fault detection algorithm using the selected mother wavelet (db1) exhibits a reputable correlation with the fault signal. The main aim of the suggested fault detection algorithm is to effectively detect, identify and discriminate between BCFs and SHIFs cases. Therefore, at sample *k*, the algorithm will execute as follows: -

- The ***M***-Index is determined by estimating the current approximation coefficients (maximum absolute values) for the three-phase current signals (*Ma*, *Mb*, and *Mc*) and the ground fault current index (*Mg*) using the mathematical formulations specified in Eq (5). In this context, the ground current approximation coefficient (maximum absolute value) is utilized specifically for ground fault classification. In Eq (5), the overall number of samples/one cycle is represented by *N*, while *n* denotes the sampling order/sliding window that covers a complete one cycle (20 ms).

$$\begin{array}{c} Ma(k)={max|A1_{ia(n)}|}_{n=k-N+1}^{k}\\ Mb(k)={max|A1_{ib(n)}|}_{n=k-N+1}^{k}\\ Mc(k)={max|A1_{ic(n)}|}_{n=k-N+1}^{k}\\ Mg(k)={max|A1_{ig(n)}|}_{n=k-N+1}^{k} \end{array}\}$$ (5)

Where: -

Maximum Absolute values (M- index values): is a mathematical concept used to quantify the absolute values of a given set of the current approximation coefficients as a maximum value that aim to capture the most significant features in the current signals (Eq (5)). In the suggested scheme, *M- values* are used to estimate the maximum absolute values of the approximate output (1^st^ approximation coefficient A1 of 0~50 kHz component) for a 1-cycle period. The sampling rate employed is 200 kHz (i.e., 4000 samples /cycle at 50 Hz). The whole process is based on a moving window approach where the one-cycle window is moved continuously by one sample. The criteria of the maximum absolute sum values to stay above the threshold level for a duration more than one power cycle, which is equivalent to more than 20 ms (>20 ms).

- Calculate Absolute Sum values (***S- values***): is a mathematical concept used to quantify the sum values of a given set of the current detail coefficients as an absolute value that aim to capture the most significant features in the current signals. The *S- values* are used to estimate the absolute sum values for the current detail coefficients (*Sa*, *Sb*, and *Sc*) of the decomposed current signals. The detail output (1^st^ detail coefficient D1 of 50~100 kHz) for a 1-cycle period. The whole process is based on a moving window approach where the one-cycle window is moved continuously by one sample., as:—The mathematical formulations in Eq (6), are then applied to determine the absolute sum for the current detail coefficients (*Sa*, *Sb*, and *Sc*) of the decomposed current signals, as: -

$$\begin{array}{c} Sa(k)=\sum_{n=k-N+1}^{k}\left|D1_{ia(n)}\right|\\ Sb(k)=\sum_{n=k-N+1}^{k}\left|D1_{ib(n)}\right|\\ Sc(k)=\sum_{n=k-N+1}^{k}\left|D1\_ic(n)\right| \end{array}\}$$ (6)

- The next step is to calculate the ***H-Index***, which is determined by comparing the absolute sum values of the current detail coefficients (*Ha*, *Hb*, and *Hc*) as follows:—

$$\begin{array}{c} Ha(k)=|Sa(k)-Sb(k)|\\ Hb(k)=|Sb(k)-Sc(k)|\\ Hc(k)=|Sc(k)-Sa(k)| \end{array}\}$$ (7)

Where: -

***H-Index*** value: is the differences of the sum absolute value for current detail coefficients (*Ha*, *Hb*, and *Hc*) of the decomposed current signals. Which the ***H-Index*** is the value represents a specific metric due to the nature of extracted feature from fault signals of the decomposed current signals using the mathematical formulations in Eq (7). The detail output (1^st^ detail coefficient D1 of 50~100 kHz) for a 1-cycle period. The whole process is based on a moving window approach where the one-cycle window is moved continuously by one sample.

- Finally, the ratios (*Ra*, *Rb*, and *Rc*) are calculated as classification parameters in the case of BCFs and SHIFs based on the approximation coefficients for the three-phase current signals follows: -

$$\begin{array}{c} Ra(k)=|Ma(k)/Mb(k)|\\ Rb(k)=|Mb(k)/Mc(k)|\\ Rc(k)=|Mc(k)/Ma(k)| \end{array}\}$$ (8)

- The previously calculated ratios (according to mathematical Eq (8)) are used for detecting BCFs and SHIFs by applying the following inequalities:
  ■ The fault is confirmed as a shunt non-HIF (Low-Impedance Fault) if any of the current approximation coefficient (***M-values***) is larger than the set threshold *Mth*:
◾Ma>MthorMb>MthorMc>Mth
  ■ The fault is confirmed as a BCF if all the coming conditions are fulfilled:

Condition-1: All current approximation coefficients (***M-values***) are less than the set threshold *Mth*

◾Ma<Mth&Mb<Mth&Mc<Mth

Condition-2: One or more of the current details coefficient (***H***-values) exceed the setting threshold *Hth*

◾Ha>HthorHb>HthorHc>Hth

Condition-3. At least one of the approximations coefficients ratios (***R***-values) is larger than the threshold *Rth*

◾Ra>RthorRb>RthorRc>Rth

■ The fault is confirmed as shunt HIF if the two aforementioned conditions (Condition-1 and Condition-2) plus the following condition (Condition-4) are fulfilled:

Condition-4: All the approximation coefficients ratios (R-values) are less than the setting threshold *Rth*

◾Ra<Rth&Rb<Rth&Rc<Rth

**Fig 5:**
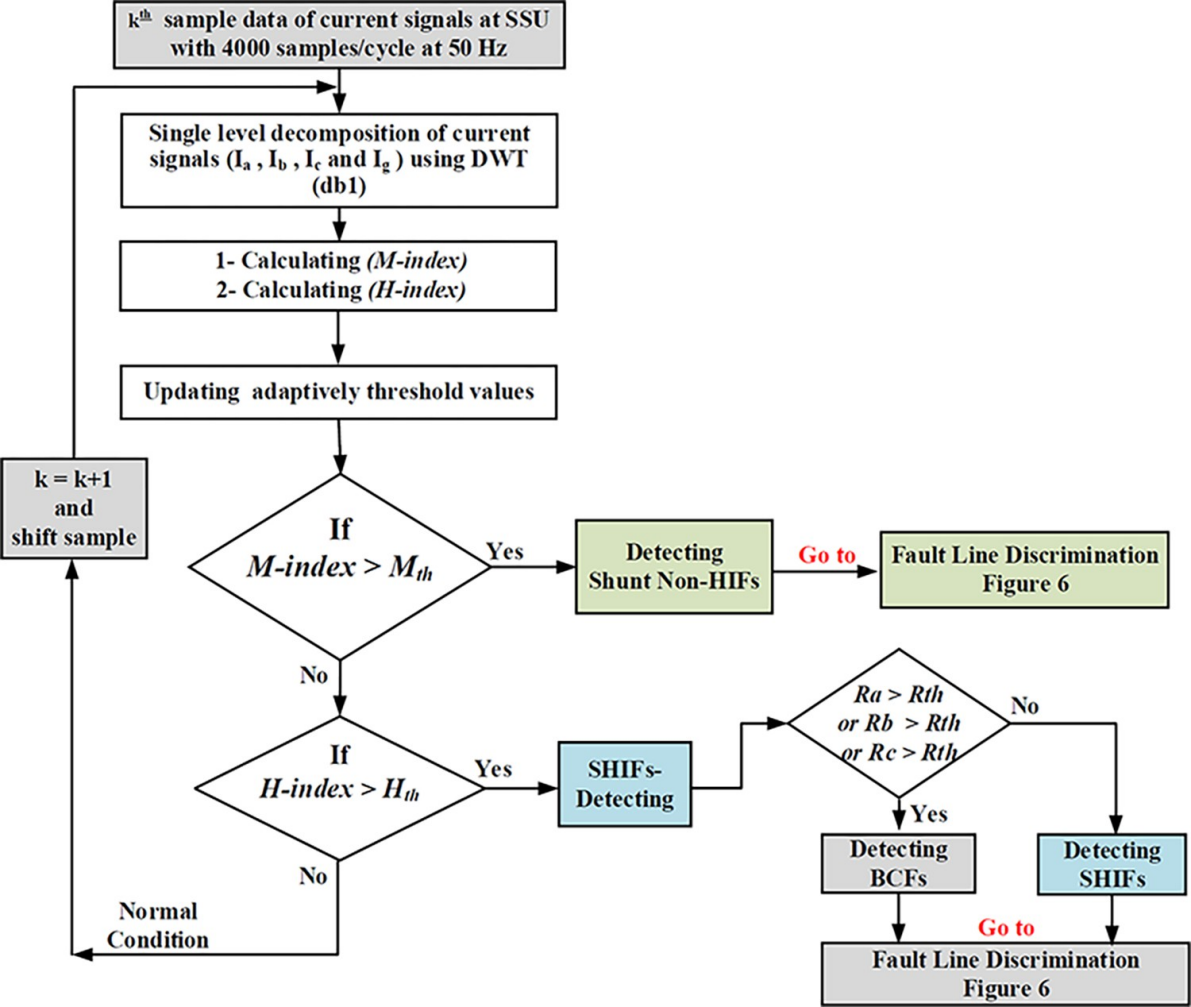
Flowchart of the proposed fault identification algorithm and calculating adaptively threshold values.

The procedure is based on a continuous one-cycle moving window approach. To ensure a proper tripping decision, it is crucial for the ***H-values*** to consistently exceed the setting threshold for a duration more than one power cycle, which is equivalent to more than 20 ms **(>20 ms**). The overall flowchart of the suggested algorithm for detecting/identifying and discriminating BCFs and SHIFs is shown in Fig 5.

The value of the setting threshold *Rth* is chosen based on the assumption that the typical value of SHIF current is in the range of 70% to 120% of the normal load current, as discussed in Ref. [22]. Therefore, *Rth* is selected in this algorithm to be a fixed setting with a value of 1.2. On the other hand, the threshold values of *Mth* (***M***-Index threshold) and *Hth* (***H***-Index threshold) are dynamically calculated and updated based on the normalized value of the pre-fault current. These setting threshold values are adjusted autonomously without any intervention from operators, as elaborated in the subsequent discussion.

#### Calculating adaptively threshold values

To handle the normal current variations, based on the deconstructed current approximation coefficient signals, the suggested scheme unit (SSU) changes the threshold values adaptively as follows: -

Mth=C1×|(Ma+Mb+Mc)/3| (9)

Hth=|(Mth×C2)| (10)

In different fault scenarios, *C*1 is preferred to express the maximum loading of the protected line and adequately distinguish between healthy and faulty phases. Moreover, for higher sensitivity, *C*2 is chosen as 5%. So, the coefficients *C*1 and *C*2 are selected to have numerical values of 1.3 and 5%, respectively. In order to validate its capability across various system topologies, the adaptive changes in threshold settings are examined in various configurations to ensure their effectiveness. The choice of the coefficient *C*1 margin value by 30% increase must be noted as an empirical value for the system under consideration.

The current approximation coefficients are calculated every cycle (*M*recent-values) and compared against the previous, current approximation coefficients values (*Mprevious* -values). When there is a considerable difference (|*Mrecent*−*Mprevious*|>Δ*M*), and simultaneously, the no-fault event is detected, and a sustained variation is ensured. When the new load value is settled for 1.5 consecutive cycles, the scheme will update its threshold values (*Mth*, *and Hth*). Practically, the load change sensitivity Δ*M* is determined by the operator. It is chosen here for the system under study as 15% (Δ*M* = 0.15).

### Faulty line discrimination algorithm

In interconnected networks, the BCFs and SHIFs may be detected by more than one relay (or more than SSU). Hence, it is necessary to identify the faulty line accurately and to make sure that only the SSUs on the faulty line will respond. As illustrated for the interconnected transmission IEEE 9-bus system shown in Fig 3, at each end of the protected lines L (7–8), L (8–9), the SSU is installed without any synchronization or communication between the SSUs: SSU1, SSU3 for L (7–8) and SSU2, SSU4 for L (8–9). The fault direction and faulty zone identification can be verified by each SSU to ensure adequate selectivity, as described in this section.

For frequency-band power over 100–50 kHz (1^st^ detail), the extracted transient aspects of both current and voltage signals aerial modes are employed to decide the faulty direction. The superimposed voltage and current signals observed at each SSU are used to calculate the transient power, which is then used to determine the fault direction by checking the polarity variation of the transient power. To determine the index of transient aerial power within a specified frequency range, the sum of two consecutive power cycles is utilized. This calculation involves multiplying the first detail coefficient (*D*1) of the aerial mode for both voltage (*D*1_*Vr*_) and current (*D*1_*ir*_) at each SSU. The resulting value is derived using the following equation [33, 34], (2*N* presents the samples in a window, *n* denotes the sampling order within a sliding window that covers a complete cycle).


$$P_{D1}(k)=sign(\sum_{n=k-2N+1}^{k}\left|{D1}_{Vr}(n)\times {D1}_{ir}(n)\right|)$$ (11)

**Fig 6:**
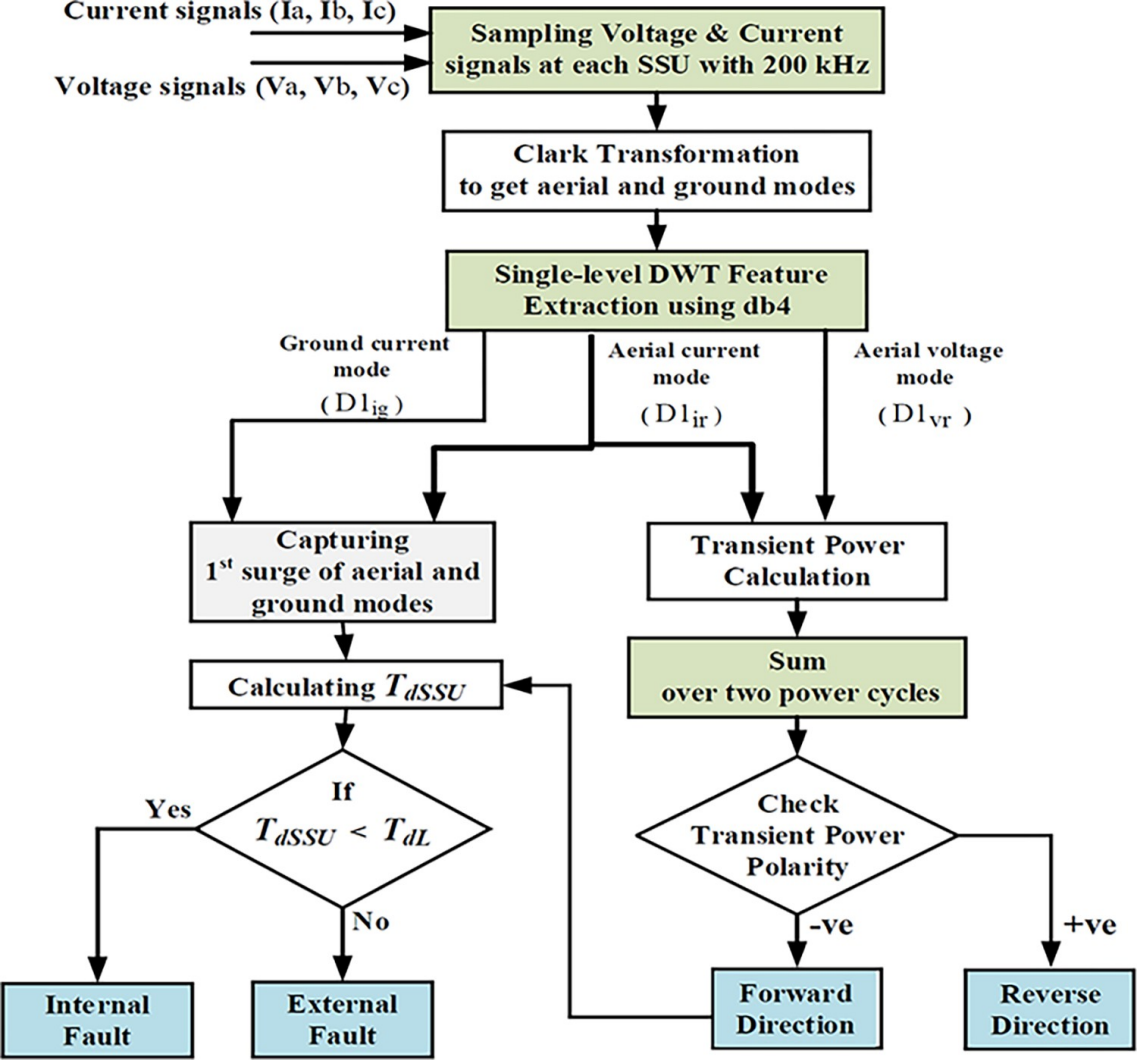
Flowchart of the faulty line discrimination algorithm.

The transient power’s polarity, represented by *P*_*D*1_(*k*), is used to verify the direction of the fault. A negative value indicates a forward direction, while a positive value indicates a reverse direction, as shown in the flowchart of Fig 6 for BCFs and SHIFs. During fault detection (forward fault), the time difference between the 1^st^ spike of current in the aerial (α)/ground (0) modes, *T*_*d*SSU_ at each SSU is compared to the pre-determined time difference for a simulated far-end fault on the protected line (*T*_*dL*_) [35]. During the relay commissioning stage, the pre-estimated time difference (*T*_*dL*_) is only calculated **ONCE** as expressed in the following equation: -

$$T_{dL}={\left|\left(Time\;of\;1st\;spike\;of\;ground\;mode\;(0))-(Time\;of\;1st\;spike\;of\;aerial\;mode\;(\alpha )\right)\right|}_{for\;a\;far-end\;fault}$$ (12)

Thus, for any SSU to confirm that the fault is internal, two conditions must be fulfilled, which can be summarized as follows:

1. The fault must be in the forward direction (Condition-5).
2. *T*_*d*SSU_<*T*_*dL*_. (Condition-6).

The **SSU,** with the two confirmed conditions, acts as primary protection. Otherwise, it acts as backup protection.

### Requirement for real-time implementation

The current transformer and voltage transformer (detectors), used in the area of capturing the transient features from the faulted signals with high-frequency range should have high bandwidth. The traditional measuring equipment, based on electrical engineering, normally has a limited bandwidth and response speed. On contrary, optical sensors and fiber-optic sensors can be used in such applications as they have primary advantages over traditional sensors include: high accuracy, wide dynamic range, high bandwidth, reduced size and weight, safe and environmentally friendly, low maintenance. A fiber-optics scheme is widely used for long TLs due to its high speed (1 Tb/s) [36].

To implement the proposed protection scheme in real-world applications, specialized hardware is needed. This hardware is responsible for sampling the output of the current transformer and voltage transformer, conducting online processing of the information, detecting and classifying faults, and executing the required commands. For practical implementation, it is crucial to sample the signal at a frequency of 200 kHz and convert it into equivalent binary information with a precision of 16 bits [37]. After the binary information is obtained, it will be transmitted to a 32-bit processor. Additionally, it is essential to transfer the information derived from the processing and decision-making to the output ports. The primary CPU needed for implementing the proposed strategy is an AT91SAM7 × 256 microcontroller. This microcontroller, specifically Microchip’s ARM-based SAM7S256, belongs to the SAM7S series of flash microcontrollers and is based on the 32-bit ARM7TDMI RISC processor.

## Testing the performance of the suggested scheme unit (SSU)

As discussed, SSU is designed to be installed in interconnected transmission systems to detect and isolate BCFs which are considered a challenge for conventional distance relays. Using ATP/EMTP, the IEEE 9-bus system of 230 kV shown in Fig 3 is simulated to model different BCFs types and to evaluate the performance of SSU, ATP/EMTP Model and data are given in S1 File. The main parameters of the IEEE 9-bus system are given in Refs. [38, 39]. The SSU is subjected to testing under various fault conditions:

■ Different types of BCFs (BCF-T1, BCF-T2, and BCF-T3 displayed in Fig 1). For BCF-T2 and BCF-T3, one end is connected to the earth after the phase is broken (opened) by 35 ms. The earthed end is examined with both high and low fault resistance.
■ Different types of SHIFs.
■ All the aforementioned types are examined at numerous fault locations and fault inception angles on the current waveform covering the protected line.

The following subsections examine the performance of SSU for BCFs, and SHIFs detection during different fault conditions. The capability of SSU for faulty line discrimination and changing threshold values adaptively is also investigated. Finally, the general features of the suggested scheme will be summarized.

### Broken conductor faults detection

**Fig 7:**
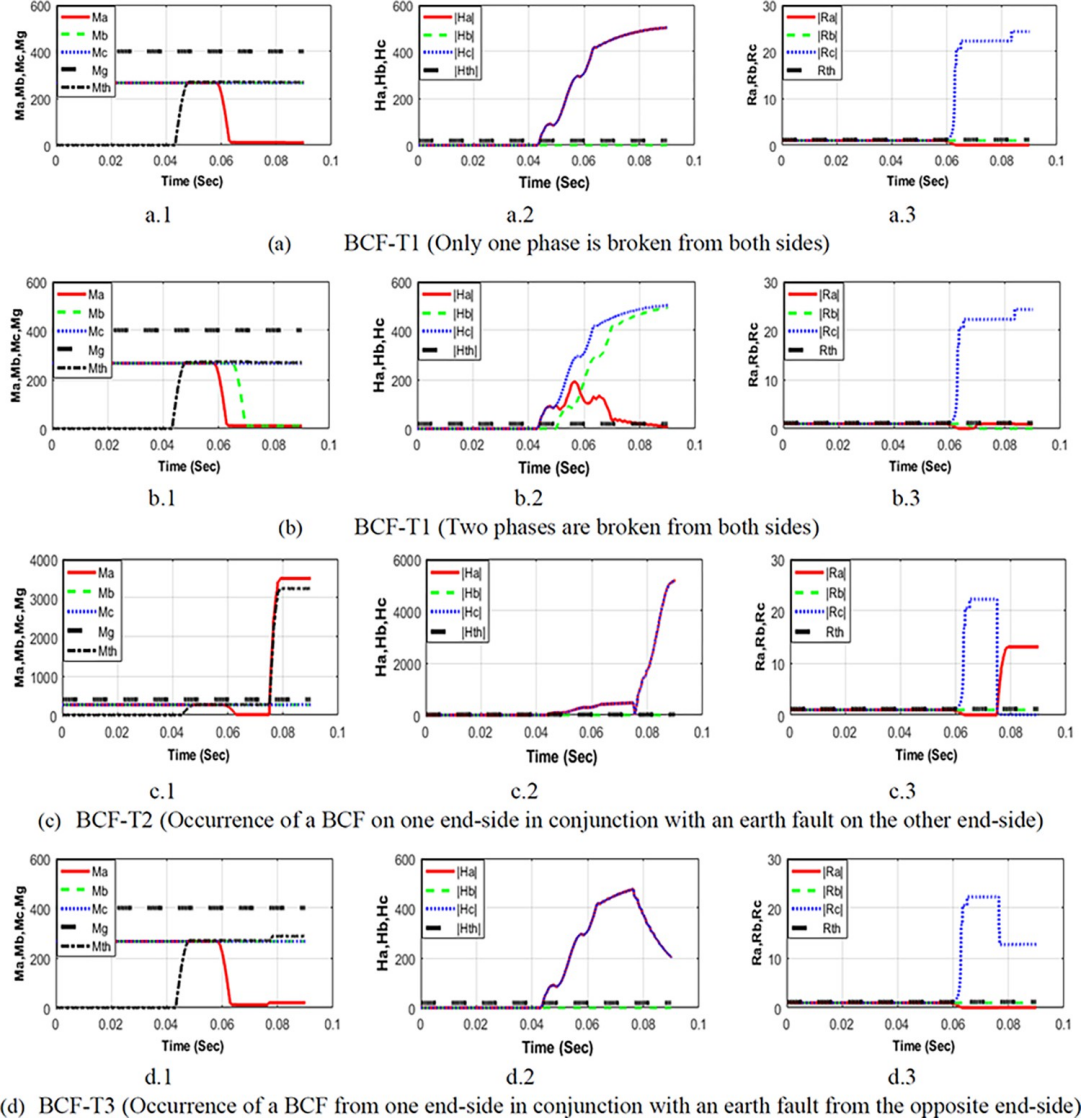
Calculated (*M*−*values*), (*H*−*values*), and (*R*−*values*) for BCFs at 20 km from SSU1.

To confirm the performance of SSU in detecting different BCFs types, the performance of the SSU1 installed on line L (7–8) is calculated for different simulated BCFs occurring at 20 km from SSU1, as illustrated in Fig 7. The figure demonstrates the curves representing the variation of current approximation coefficients values ***M-values*** (*Ma*, *Mb*, *Mc*, *Mg*), The difference between the sum of the absolute values ***H-values*** for current detail coefficients (*Ha*, *Hb*, *Hc*), and ***R-values*** (*Ra*, *Rb*, and *Rc*), which denotes the ratios of approximation coefficients.

Fig 7(A) represents the performance of the SSU1 for a BCF from both sides (BCF-T1); it is evident from the figure that:

- All the *M-values* in Fig 7(A.1) are less than the threshold *Mth* (Condition-1),
- At least one of the *H-values* (*Ha and Hc* in Fig 7(A.2)) exceeds the setting *Hth* (Condition-2) and,
- At least one of the *R-values* (*Rc* in Fig 7(A.3)) exceeds the setting *Rth* (Condition-3).

According to the conditions stated in Section **Fault Detection Algorithm**, the fault is correctly confirmed as a BCF. Hence, the second algorithm will be activated to decide the faulty line. Fig 7(B) also illustrates another example of BCF-T1 but with two open phases from both sides simultaneously at 0.04 s. Again, the three conditions are fulfilled, as shown in the figure, and SSU1 detects such faults correctly.

Fig 7(C) and 7(D) display the calculated features for the two other BCFs types (BCF-T2 and BCF-T3), where one terminal is earthed with high fault resistance (50 ohms). It is evident from the figures that:

- All the *M-values* in Fig 7(C.1) and 7(D.1) are less than the threshold *Mth* (Condition-1),
- At least one of the *H-values* (*Ha and Hc* in Fig 7(C.2) and 7(D.2)) exceeds the setting *Hth* (Condition-2) and,
- At least one of the *R-values* (*Rc* in Fig 7(C.3) and in 7(D.3)) exceeds the setting *Rth* (Condition-3). Hence, both faults are also confirmed as BCFs.

### Shunt high impedance fault detection

**Fig 8:**
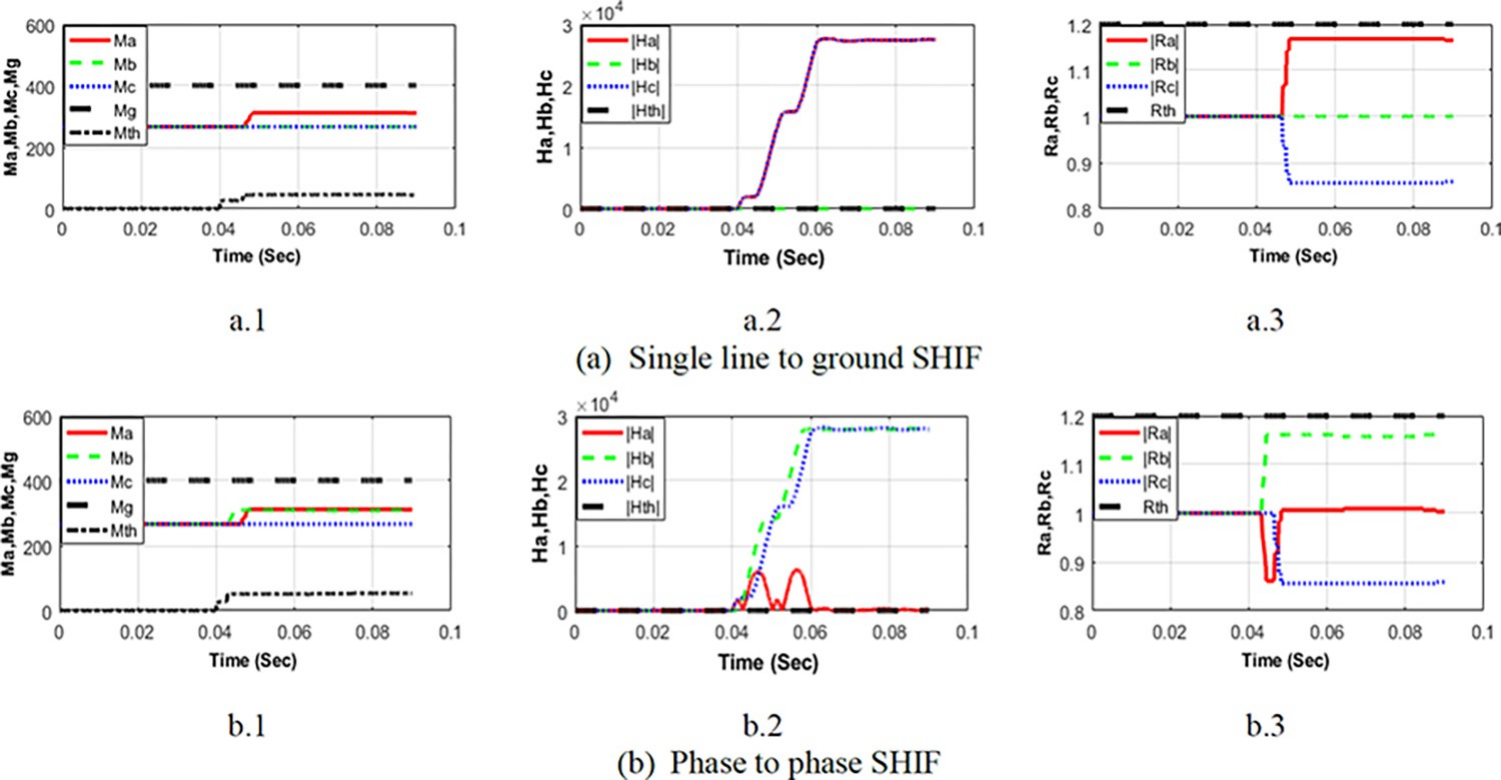
Calculated (*M*−*values*), (*H*−*values*), and (*R*−*values*) for different SHIFs at 20 km from SSU1.

Shunt HIFs (SHIFs) are also simulated to examine the proposed scheme’s effectiveness for detecting SHIFs. Fig 8 displays the combined calculated features for low and high-frequency components used to enhance the detection of SHIFs. Fig 8(A) represents a single line to ground HIF, where Fig 8(B) shows the results for a phase-to-phase HIF. The three criteria (Condition-1, Condition-2, and Condition-4) are satisfied in both cases. To illustrate, in the case of a single line-to-ground shunt high-impedance fault (SLG-SHIF):

- All the ***M-values*** (Fig 8(A.1)) are less than the threshold *Mth* (Condition-1),
- At least one of the ***H-values*** (Fig 8(A.2)) exceeds the setting *Hth* (Condition-2) and,
- All the ***R-values*** (Fig 8(A.3)) are less than the setting limit *Rth* (Condition-4).

### Impact analysis of different fault conditions (types, location, fault resistance, and inception angle)

For the tested interconnected IEEE 9-bus system, SSUs installed at each end of the protected lines L (7–8) and L (8–9) are extensively tested for different fault conditions. As demonstrated in Table 2, simulated faults are correctly detected and discriminated for various BCF types and SHIFs types. In all cases, the fault type is correctly detected within only 24.5 ms after fault occurrence. The results demonstrate that the SSU exhibits immunity to various fault types, location, fault resistance and inception angles.

**Table 2:** Samples for simulation results of various BCFs and SHIF types for tested interconnected IEEE 9-bus system.

Simulated case	Line No.	Location (%)	Fault resistance (Ω)	Fault time occurrence, (sec)	The maximum absolute *M-values* and the difference absolute sum *H*-values of the detailed coefficients				Approximation coefficients ratios *R-values*			Fault classification	Fault detection time (ms)
					|*Ma*| ×10^3^ |*Ha*|×10^3^	|*Mb*|×10^3^ |*Hb*|×10^3^	|*Mc*|×10^3^ |*Hc*|×10^3^	|*Mg*| ×10^3^	|*Ra*|	|*Rb*|	|*Rc*|		
BCF-T1 One phase	Line (7–8)	5	* 10000	0.05	0.003	0.267	0.267	0.268	0.011	1.000	89.00	BCF	21.23
					0.512	0.020	0.512						
BCF-T1 One phase		95	* 10000	0.0594	0.064	0.267	0.267	0.287	0.239	1.000	4.172	BCF	22.08
					0.327	0.029	0.327						
BCF-T3 Low resistance		15	10	0.045	0.0100	0.267	0.267	0.27	0.037	1.000	26.69	BCF	24.05
					0.452	0.019	0.452						
BCF-T3 High resistance		85	50	0.047	0.073	0.267	0.267	0.284	0.273	1.000	3.657	BCF	23.78
					0.266	0.017	0.266						
BCF-T2 Low resistance		20	10	0.049	0.012	0.267	0.267	0.27	0.045	1.000	22.25	BCF	21.28
					0.447	0.012	0.447						
BCF-T2 High resistance		98	50	0.052	0.066	0.267	0.267	0.274	0.247	1.000	4.045	BCF	22.09
					0.337	0.015	0.337						
BCF-T1 Two-phases		20	* 10000	0.04	`0.012	0.016	0.267	0.272	0.750	0.059	22.25	BCF	21.24
					0.034	0.417	0.451						
BCF-T1 Two-phases		97	* 10000	0.05	0.065	0.057	0.267	0.29	1.140	0.213	4.108	BCF	22.11
					0.045	0.360	0.315						
SHIF-(SLG)		20	280	0.055	0.311	0.267	0.267	0.045	1.165	1.000	0.859	SHIF	20.88
					27.969	0.010	27.969						
SHIF-(SLG)		95	280	0.04	0.307	0.267	0.267	0.04	1.149	1.000	0.869	SHIF	21.22
					17.671	0.010	17.671						
SHIF-(L-to-L)		30	280	0.051	0.308	0.307	0.267	0.05	1.003	1.149	0.867	SHIF	21.55
					0.013	27.404	27.417						
SHIF-(L-to-L)		85	280	0.043	0.304	0.303	0.267	0.048	1.003	1.134	0.878	SHIF	20.89
					0.291	36.044	36.335						
BCF-T1 One phase	Line (8–9)	10	* 10000	0.055	0.011	0.237	0.237	0.239	0.046	1.000	21.55	BCF	21.20
					0.400	0.011	0.400						
BCF-T1 One phase		90	* 10000	0.0594	0.090	0.237	0.237	0.24	0.379	1.000	2.633	BCF	22.26
					0.175	0.011	0.175						
BCF-T3 Low resistance		23	18	0.04	0.021	0.237	0.237	0.23	0.088	1.000	11.29	BCF	23.88
					0.368	0.011	0.367						
BCF-T3 High resistance		88	45	0.046	0.087	0.237	0.237	0.24	0.367	1.000	2.724	BCF	24.09
					0.166	0.013	0.166						
BCF-T2 Low resistance		50	13	0.044	0.049	0.237	0.237	0.236	0.207	1.000	4.837	BCF	22.08
					0.235	0.010	0.235						
BCF-T2 High resistance		96	55	0.048	0.095	0.237	0.237	0.24	0.401	1.000	2.495	BCF	22.20
					0.156	0.012	0.156						
BCF-T1 Two-phases		12	* 10000	0.043	0.014	0.013	0.237	0.227	1.077	0.055	16.93	BCF	22.18
					0.013	0.387	0.374						
BCF-T1 Two-phases		75	* 10000	0.053	0.078	0.073	0.237	0.24	1.069	0.308	3.038	BCF	23.07
					0.001	0.180	0.179						
SHIF-(SLG)		10	280	0.055	0.260	0.237	0.237	0.028	1.097	1.000	0.912	SHIF	20.78
					13.72	0.012	13.72						
SHIF-(SLG)		90	280	0.041	0.246	0.237	0.237	0.012	1.038	1.000	0.963	SHIF	21.26
					24.798	0.011	24.798						
SHIF-(L-to-L)		21	280	0.054	0.246	0.237	0.237	0.012	1.038	1.000	0.963	SHIF	21.08
					25.14	0.013	25.14						
SHIF-(L-to-L)		88	280	0.043	0.246	0.247	0.247	0.017	0.996	1.042	0.963	SHIF	20.88
					0.045	7.474	7.519						
Where: *10000 Ω denotes Brocken conductor from both sides, For Line (7–8): *Mth* = 0.401×10^3^, *Hth* = 0.020×10^3^ and *Rth* = 1.2, While for Line (8–9): *Mth* = 0.356×10^3^, *Hth* = 0.018×10^3^ and *Rth* = 1.2													

### Testing SSU capability for faulty line discrimination

For a BCF of type-1 (BCF-T1) at F2 in line L (8–9) shown in Fig 3, the fault is detected by all SSUs at lines L (7–8) and L (8–9) as the three conditions (Condition-1, Condition-2, and Condition-3) are all fulfilled in the SSUs of the two lines. To distinguish between forward and reverse faults, each SSU utilizes the Transient Power Direction Technique (TPDT). This technique allows the SSU to analyze transient power flow patterns and differentiate between forward and reverse faults. In the context of fault identification, a fault is correctly classified as a forward fault if the direction has a negative sign. Conversely, a fault is identified as a reverse fault if the direction has a positive sign. This sign convention enables accurate determination and categorization of fault direction based on the results obtained through the Transient Power Direction Technique utilized by SSU. The SSU is enabled only for forward faults and will be blocked for reverse faults. To ensure proper coordination in the event of a forward fault, each SSU should determine the fault zone to select the appropriate operational time. Upon detecting a forward fault, the calculated *T*_*d*SSU_ is compared locally with the corresponding *T*_*dL*_ at each individual SSU for decision-making processes. The two conditions presented in Section Faulty Line Discrimination Algorithm must be fulfilled for internal faults.

In this section, the selectivity of SSU in the interconnected network is extensively examined. Faults are applied in two adjacent lines in the tested IEEE 9-bus system shown in Fig 3(F1 and F2) to examine the ability of different installed SSUs to discriminate between internal and external faults using condition-5 and condition-6 presented in Section **Faulty Line Discrimination Algorithm**. The results are summarized in Table 3, where several BCFs in both lines L (7–8) and L (8–9) are simulated with different inception angles and fault positions over each protected line. As illustrated, (F) and (R) denote the faults in the forward and reverse directions. Referring to Table 3, as an example, for a BCF at F2 located at 10% of the line length, the fault is correctly classified as a forward fault in SSU1, SSU2, and SSU4, while SSU3 is blocked since it correctly classifies such fault as a reverse fault. At the same time, *T*_*d*SSU2_<*T*_*dL*8−9_ and *T*_*d*SSU4_<*T*_*dL*8−9_, which means both condition-5 and condition-6 in Section The faulty Line Discrimination Algorithm is fulfilled, and the fault is classified as an internal BCF for SSU2 and SSU4. Both SSU2 and SSU4 act as primary protection. On the other hand, T_dSSU1_>T_dL7−8_ and thus SSU1 correctly discriminates such fault as an external forward fault (condition-5 is only fulfilled), and thus SSU1 acts as backup protection. The results indicate that the SSU, when used in interconnected networks, exhibits high reliability and adequate sensitivity as both primary and backup protection. It exhibits immunity towards variations in the fault inception angle and position.

**Table 3:** Results for faulty line discrimination for BCFs in lines L (7–8) and L (8–9).

BCFs conditions			Performance of different SSUs											
Fault Position	Location (%)	Inception Angles (φ°)	SSU1			SSU3			SSU2			SSU4		
			Power Direction	*T*_*d*SSU1_ μs	*T*_*dL*7−8_ μs	Power Direction	*T*_*d*SSU3_ μs	*T*_*dL*7−8_ μs	Power Direction	*T*_*d*SSU2_ μs	*T*_*dL*8−9_ μs	Power Direction	*T*_*d*SSU4_ μs	*T*_*dL*8−9_ μs
F1 in line L (7–8)	10	0	-0.816 (F)	2.01	19.99	-1.492 (F)	17.99	19.99	1.493 (R)	18.29	79.99	-0.073 (F)	97.98	79.99
	30	90	-2.012 (F)	5.06		-2.005 (F)	14.94		2.006 (R)	16.82		-0.150 (F)	94.94	
	60	180	-3.238 (F)	12.73		-6.961 (F)	7.27		6.962 (R)	9.288		-0.194 (F)	87.27	
	92	216	-2.367 (F)	18.01		-2.422 (F)	1.989		2.422 (R)	3.979		-0.207 (F)	81.99	
F2 in line L (8–9)	10	0	-0.046 (F)	25.99		0.248 (R)	5.00		-0.248 (F)	4.899		-1.169 (F)	75.10	
	30	90	-1.069 (F)	50.01		5.068 (R)	31.87		-5.069 (F)	29.87		-12.71 (F)	50.13	
	60	180	-0.146 (F)	80.37		1.834 (R)	52.58		-1.834 (F)	50.08		-2.182 (F)	29.92	
	92	216	-0.212 (F)	90.5		2.462 (R)	76.78		-2.462 (F)	74.79		-4.066 (F)	5.209	

### Testing SSU capability for changing threshold values adaptively

**Fig 9:**
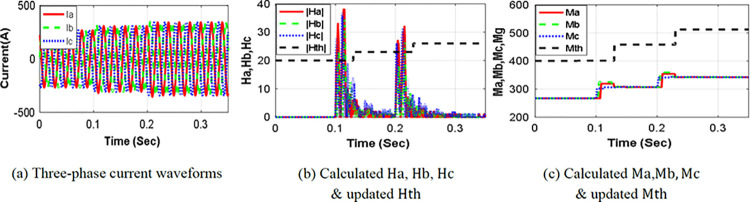
Case of normal load changing.

This section analyzes the performance of SSU during normal load changes. Specifically, Fig 9(A) presents the current waveforms detected at SSU1, which is located at the beginning point of the protected line L (7–8). The waveforms depict the detected currents during normal load increments occurring at 0.100 sec and 0.200 sec, respectively. Fig 9(B) and 9(C) exhibit the ***H-index values*** (*Ha*, *Hb*, *Hc*) and the ***M-index values*** (*Ma*, *Mb*, *Mc*), for normal load changes. In this case, no fault is detected because none of the ***H-index values*** exceed the threshold setting values for more than one cycle. Consequently, the threshold setting values are updated to reflect the absence of fault conditions, and to confirm the normal load change. This adaptive updating ensures that the thresholds remain adjusted to the current operating conditions. The scheme performance shows that the significant change in ***M-index values*** is accurately interpreted as load increases. Therefore, the threshold values *Mth*, *and Hth* are adjusted to new setting values, based on Eq (9) and Eq (10), as illustrated in Table 4. The new threshold setting values is automatically determined based on the updated normalized value of the ***M-index values***, eliminating the requirement for human intervention. This automated process ensures that the threshold adapts to the current conditions, allowing for accurate and reliable normal load change without manual adjustments.

**Table 4:** Changing threshold values adaptively during normal load changing.

Simulated case	Line No.	Location (%)	Switching Time occurrence, (sec)		The maximum absolute *M-values* and the difference absolute sum *H*-values of the detailed coefficients				Threshold values		Threshold values event
			From	To	|*Ma*| ×10^3^ |*Ha*|×10^3^	|*Mb*|×10^3^ |*Hb*|×10^3^	|*Mc*|×10^3^ |*Hc*|×10^3^	|*Mg*| ×10^−3^	(*Mth* & *Hth*) ×10^3^		
Normal Case	Line (7–8)	10	0.00	0.100	0.280	0.280	0.280	0.268	0.401	0.020	Adaptive
					0.012	0.012	0.012				
Normal Load changing (1)		10	0.100	0.020	0.301	0.301	0.301	0.287	0.480	0.024	Adaptive
					0.015	0.015	0.015				
Normal Load changing (2)		10	0.020	0.036	0.350	0.350	0.350	0.270	0.520	0.028	Adaptive
					0.018	0.018	0.018				

## General features of the suggested scheme

To validate the superiority of the Suggested Scheme, a comparative study was carried out, contrasting its performance against other existing schemes documented in the literature are presented in Table 5. This study aimed to assess and demonstrate the advantages, effectiveness, and superiority of the suggested scheme over alternative methods proposed in prior research that support the suggested scheme ’s superiority in terms of various performance metrics and criteria. Table 5 presents the results of this study, which categorized the schemes used in the comparison as follows: -

The schemes utilized in the comparative study can also be categorized according to the following classification criteria:

- Schemes applied only for BCFs detection are outlined in Refs.: [13–15, 17–19, 21].
- Schemes applied for BCFs location are outlined in Ref. [12].
- Schemes that were used for both the detection and location of BCFs are outlined in Ref. [20],
- Schemes applied only for SHIFs detection are outlined in Refs.: [10, 22, 24].

**Table 5:** Comparison of the suggested scheme with published schemes.

Item		General requirements			Fault detection features					Tested system		
		Measurements taken	Need for applying AI techniques	Need for Communications	Applied approach	Threshold values	OCFs Types Detected	SHIFs Types Detected	Faulty Line Discrimination	Transmission/Distribution	System level voltage	Simulations via
Suggested scheme		1/end	x	x	DWT	Adaptive	BCF-T1 & BCF-T2 & BCF-T3	√	√	T	IEEE 9-bus, 230 kV	ATP
**[12]**	Gilany et al. (2010)	1/end	√	√	ANN	ــــ	BCF-T1	x	x	T	220 kV	MATLAB
**[13]**	Hasaneen BM et al. (2016)	1/end	√	x	ANN	ــــ	BCF-T1	x	x	T	220 kV	MATLAB
**[14]**	Chandra et al. (2016)	1/end	x	x	I_2_ sequence current	Fixed	BCF-T1	x	x	T	138 kV	MATLAB
**[15]**	Al-Baghd. et al. (2022)	1/end	x	x	I_2_ / I_1_ sequence current	Fixed	BCF-T1 BCF-T2 & BCF-T3	x	x	D	33/11 kV	MATLAB
**[17]**	Koley E et al. (2014)	1/end	√	x	ANN	ــــ	BCF-T1	x	x	T	138 kV	MATLAB
**[18]**	Shukla (2017)	1/end	√	√	DWT + NBC	ــــ	BCF-T1	x	x	T	138 kV	MATLAB
**[19]**	Aleena et al. (2016)	1/end	√	x	FIS	Fixed	BCF-T1	x	x	T	220 kV	MATLAB
**[20]**	Vieira et al. (2019)	1/end	x	√	Voltage unbalance	Fixed	BCF-T1 & BCF-T2 & BCF-T3	x	x	D	13.8 kV	MATLAB
**[21]**	Adewole et al. (2020)	1/end	x	x	Current imbalance	Fixed	BCF-T1	x	x	D	69/138 kV	PSCAD
**[22]**	Adly AR et al. (2016)	1/end	x	x	DWT	Adaptive	x	√	x	T	500 kV	ATP
**[10]**	Biswal et al. (2023)	1/end	√	x	DT-CWT + KNN+SVM	ــــ	x	√	x	D	25/0.575 kV	PSCAD
**[24]**	Sangeet et al. (2023)	1/end	x	x	DWT	Fixed	x	√	x	D	IEEE 33 kV	PSCAD
Where: (ـ) = Not Reported; FIS = Fuzzy Inference System; NBC = Naive Bayes Classifier; ANN = Artificial Neural Network; KNN = K-Nearest Neighbour; DWT = Discrete Wavelet Transform, BCF-T1 = Broken TL conductor from both sides; BCF-T2 = Occurrence of a BCF on one side in conjunction with an earth fault on the other side.; BCF-T3 = Broken TL conductor located on the relay side while it is on the opposite side in BCF-T2; SHIF = shunt high impedance faults. T = Transmission system; D = Distribution system.												

A comparative summary between the suggested scheme and the most closed schemes in the literature is presented in Table 5. Ref. [10], depends on DT-CWT and Data mining technique will provide the fail detection of BCF fault, and therefore unable to distinguish between OCFs and SHIFs, also does not offer a solution for faulty line discrimination. Refs. [12–19], depends on artificial intelligence approaches will provide the fail detection of SHIF fault, and fail to distinguish between OCFs and SHIFs, Moreover, the effectiveness of these schemes is severely reliant on their architecture and training, and Refs. [14, 15], depends on the sequence components approaches with several threshold values will provide the fail to distinguish between OCFs and SHIFs, also it fails to achieve precise selectivity of the faulty line. Ref. [20], depends on voltage unbalance approach will provide the fail detection of SHIF fault, and does not offer a solution for faulty line discrimination. Ref. [21], depends on the current detection method will provide the fail detection of SHIF fault, and fail to distinguish between OCFs and SHIFs, and it does not provide a selectivity of the faulty line, and Ref. [22], depend on approximate and detail coefficients using wavelet transform will provide the mail detection of the BCF fault and see it as HIF leads to miss fault location schemes, also does not offer a solution for faulty line discrimination. Ref. [24], depends on artificial intelligence approaches will provide the fail detection of BCFs fault, and fail to distinguish between OCFs and SHIFs, Moreover, it required a large amount of training effort for good performance. The suggested scheme will provide the accurate detection of all BCFs because it depends on the high- and low-frequency components using discrete wavelet transform, and distinguishing various types of OCFs and SHIFs, Furthermore, offer a solution for faulty line discrimination.

By carefully examining the achieved comparison, and after meticulous analysis, it can be deduced and succinctly summarized that the **suggested scheme benefits** over previous schemes as follows:

- The suggested approach has demonstrated better performance than previous schemes in accuracy and efficiency, leading to novel solutions to existing problems.
- The suggested scheme is a comprehensive solution designed to identify and detect all types of Broken Conductor Faults (BCFs). Additionally, it possesses the capability to detect and identify all types of Shunt High Impedance faults (SHIFs) with equal effectiveness.
- The ability of the suggested scheme to accurately identify/distinguish all types of BCFs and shunt high impedance faults (SHIFs) based on the feature extraction of DWT in interconnected transmission systems.
- By using the transient power polarity and the time difference between the initial spikes of aerial/ground modes of current signals, the suggested scheme successfully achieves precise selectivity of the faulty line in interconnected transmission systems.
- The suggested scheme adopts a deterministic approach, avoiding the utilization of artificial intelligence (AI) approaches such as FLC, ANN, KNN, SVM, and others. This approach eliminates the requirement for training in knowledge representation and mitigates the potential drawbacks associated with AI-based protection schemes, which can be influenced by their architectural limitations and exhibit lower reliability.
- The proposed scheme relies on measurements taken only at its own end, reducing communication’s cost and complexity.
- The adaptive determination of threshold values is based on normalizing the approximation coefficients of the pre-fault current without operator interference.
- The suggested scheme performs effectively, reliably, and robustly under various fault conditions, especially for BCFs, and can perfectly distinguish between BCFs and SHIFs.
- The suggested scheme demonstrates adequacy and robustness when confronted with diverse fault scenarios, encompassing various types of Broken Conductor Faults (BCFs) such as BCF-T1 (Broken TL conductor from both sides), BCF-T2 (occurrence of a BCF on one side in conjunction with an earth fault on the other side), BCF-T3 (Broken TL conductor located on the relay side while it is on the opposite side in BCF-T2), as well as Shunt High Impedance Faults (SHIFs).
- All BCF, and SHIF types are correctly detected within only 24.5 ms after fault occurrence.
- Finally, the suggested scheme may be more flexible, reliable, and adaptable to various fault conditions, allowing for greater versatility and applicability.

## Conclusions

Detecting and accurately identifying broken conductor faults (BCFs) in high-voltage interconnected transmission systems can pose a significant challenge when relying exclusively on data collected from one end. However, a novel scheme has been presented to address this issue that employs discrete wavelet transform (DWT) to detect, distinguish, and identify accurately broken conductor faults (BCFs) and shunt high impedance faults (SHIFs) in HV interconnected transmission systems under varying fault conditions. The suggested scheme unit (SSU) significantly reduces communication costs by utilizing measurements from only one end of the line. The DWT applied to the current signals provides low and high-frequency components and discriminative features for BCFs and SHIFs detection and classification. Moreover, the SSU adaptively determines the threshold values without operator intervention or assumptions. The mother wavelet db1 is correctly selected for applying such an algorithm upon conducting an extensive comparison study among different orders. The accurate selectivity of the faulty line in the interconnected system is achieved as the SSU correctly decides the fault direction, either forward or reverse, by analyzing the polarity variation of the transient power estimated for a definite frequency band of 100–50 kHz and then discriminates internal/external faults using the time difference between the aerial and ground modes of the current signals at each SSU.

The behavior of the SSU has been extensively investigated on the lines of the interconnected IEEE 9-bus system of 230 kV for evaluating the capability of detecting BCFs or SHIFs accurately and judging the proficiency for faulty line discrimination and proper selectivity. To evaluate the performance of SSU, comprehensive simulation tests were conducted. The results indicated that SSU is immune to detecting/distinguishing various types of BCFs (traditional distance relays cannot detect OCFs at all) and SHIFs within only 24.5 ms after fault occurrence. Furthermore, the proposed scheme achieves accurate selectivity of the faulty line in multi-terminal lines by utilizing transient power polarity and the time difference between the initial spikes of aerial/ground modes of current signals. The high level of accuracy achieved by SSU is very promising. A comparative study was also conducted against other published schemes, which confirmed the superiority of the developed SSU scheme in terms of its performance. These findings attest to the reliability and robustness of the SSU under various fault scenarios in interconnected transmission systems.

## Supporting information

S1 File ATP/EMTP model and data. (RAR)
